# Bioinspired Coronary Stents: A Technological Perspective on Exosome-Mimetic Nanoengineering and Mini-Review of Existing Platforms

**DOI:** 10.31083/RCM42781

**Published:** 2025-10-23

**Authors:** Rasit Dinc, Nurittin Ardic

**Affiliations:** ^1^INVAMED Medical Innovation Institute, New York, NY 10007, USA; ^2^Med-International UK Health Agency Ltd, LE10 0BZ Leicestershire, UK

**Keywords:** coronary artery disease, exosome-mimetic nanovesicle, regenerative cardiovascular therapy, biologically inspired coatings, exosome-eluting stent

## Abstract

Coronary artery disease (CAD) remains a leading cause of morbidity and mortality worldwide. Percutaneous coronary intervention (PCI) represents the standard treatment for CAD; however, significant challenges, such as in-stent restenosis, late thrombosis, and delayed endothelial healing, remain issues for long-term outcomes. The evolution of stents from bare metal and drug-eluting platforms to bioabsorbable and nanoengineered designs has reduced, but not eliminated, these complications. Meanwhile, exosome-mimetic nanovesicle (EMNV)-coated stents have emerged as a potential approach to address these limitations since EMNVs mimic the structure and biological function of natural exosomes. This mimetic ability enables targeted delivery of therapeutic agents such as microRNAs, growth factors, and anti-inflammatory molecules. Indeed, preclinical studies have previously demonstrated the ability of these stents to reduce neointimal hyperplasia, enhance endothelialization, and modulate inflammatory responses. Engineering strategies, including stimuli-responsive release triggered by pH or enzymatic activity, further improve the precision of therapeutic delivery. However, the transition to clinical application remains in its early stages, with key obstacles including the scalability and reproducibility of EMNV production, the stability of biologic coatings during application, and regulatory classification as combination products. Therefore, clinical translation will require standardized manufacturing standards, reliable potency testing, and long-term safety studies to overcome these challenges. Personalized medicine approaches using patient-derived exosomes and artificial intelligence (AI)-assisted stent design may provide additional opportunities to accelerate the transition. This review summarizes the evolution of coronary stent technology and discusses the potential and limitations of EMNV-based platforms. This article also outlines future directions that will guide the development of EMNV-based platforms as next-generation devices in interventional cardiology.

## 1. Introduction 

Cardiovascular diseases (CVDs) remain a significant public health concern with high mortality and serious morbidity globally. Coronary artery disease (CAD) constitutes a significant portion of the burden [1]. By restoring vascular patency, percutaneous coronary intervention (PCI) has transformed the treatment of CAD. However, significant biological and mechanical limitations continue to impact long-term outcomes. To address restenosis, various endovascular intervention strategies with different platforms have been developed, from bare metal stents (BMS) to drug-eluting stents (DES) and drug-coated balloons (DCB) [2]. PCI with stent implantation remains a mainstay of therapy for revascularization in patients with atherosclerotic lesions [3].

BMS have provided mechanical scaffolding, but high rates of in-stent restenosis (ISR) due to neointimal hyperplasia (NIH) have restricted their use. Despite the introduction of DES, which has significantly reduced restenosis, the problem of restenosis has not been fully resolved. Because of their mechanism of action, the drugs used to prevent stenosis also delayed endothelialization and increased the risk of late and very late stent thrombosis (ST) [4]. Bioresorbable vascular scaffolds (BVS) that dissolve over time and are excreted by the body have been developed recently. While BVS was designed to eliminate the long-term presence of a metallic implant, concerns about scaffold thrombosis, polymer degradation, and limited radial durability have significantly limited its widespread adoption [4, 5]. These limitations highlight a fundamental challenge in developing stents that can provide sustained suppression of NIH without impairing vascular healing. Exosome-mimetic nanovesicle (EMNV) coatings offer a potential solution to address this therapeutic challenge.

Exosomes are nanometer-scale (30–150 nm) extracellular vesicles secreted by various cell types [6]. These natural exosomes play a central role in intercellular communication by delivering bioactive cargo, such as microRNAs, growth factors, and cytokines, which promote endothelial regeneration and regulate inflammation [6, 7, 8]. EMNVs are designed to mimic these vesicles and can be placed on stent surfaces to promote healing and provide site-specific delivery of anti-inflammatory signals. EMNV-covered stents aim to suppress pathological vascular remodeling while simultaneously accelerating endothelial healing. By directly targeting the biological mechanisms responsible for the late or very late thrombosis and impaired vascular healing observed in patients with DES, these stents offer the potential for a rational next step in the evolution of coronary stent technology [8]. They also support immune modulation, and provide localized and cell-specific delivery of therapeutic agents [8, 9].

This review addresses the potential of EMNV-coated stents to overcome persistent challenges in restenosis and thrombosis. It also summarizes the technological evolution of coronary stent technology from BMS to BVS and nanoengineering platforms. In this article, we also discuss the current preclinical evidence, barriers to clinical translation, and future directions for EMNV-covered stents.

## 2. Evolution of Stent Technologies

**Fig. 1.**
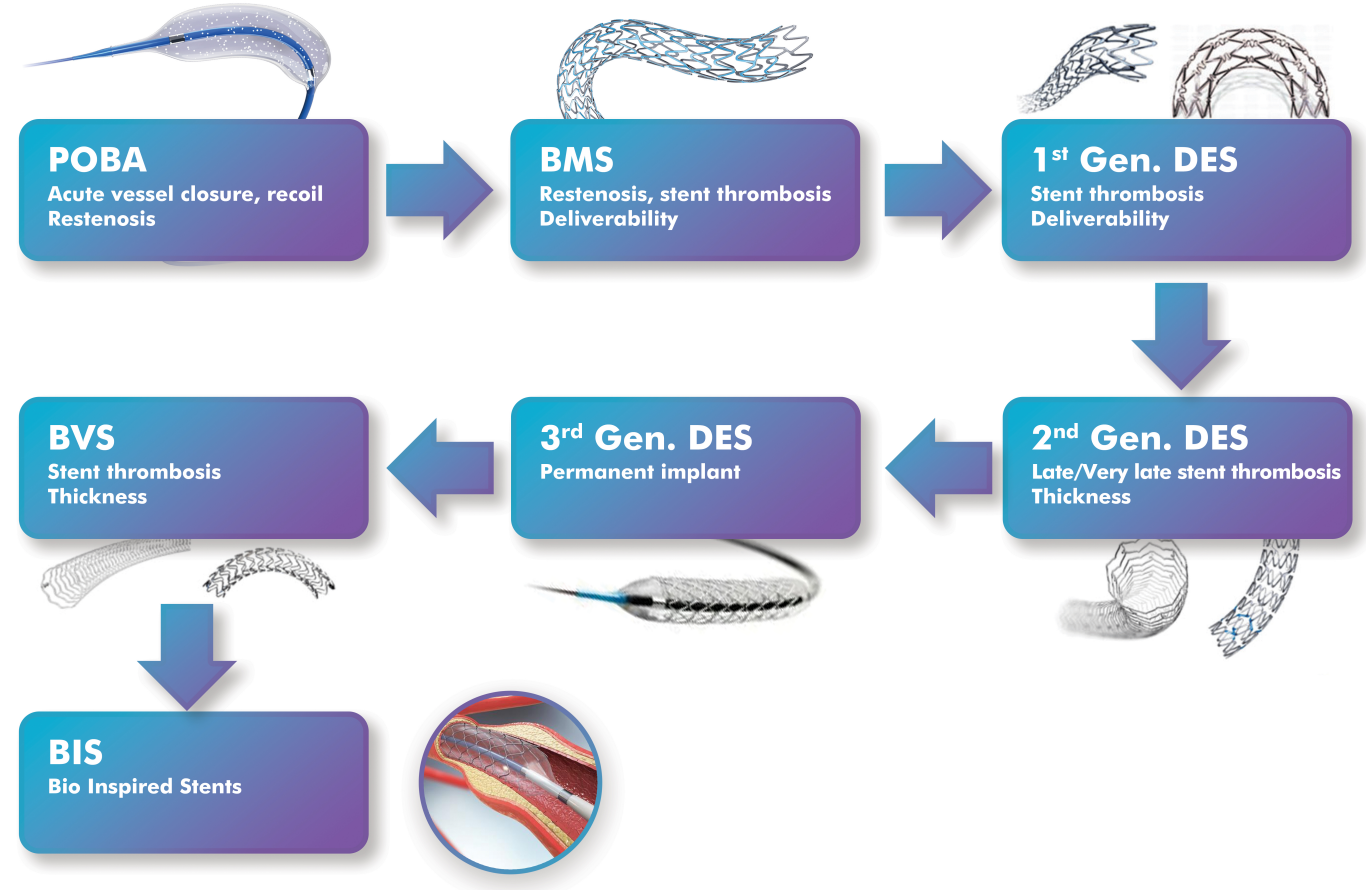
**Outlined chronological evolution of percutaneous coronary intervention technology, from POBA to emerging EMNV-coated stents**. For clarity, historical milestones based on approximate introduction or large study reports are also included below. POBA, first clinical use (1977); BMS, first clinical use (1986) and widespread adoption (1990s); first-generation DES, sirolimus (2002) and paclitaxel (2004); second-generation DES, everolimus and zotarolimus (2008 onward); BVS, Absorb BVS CE mark (2011), FDA approval (2016), and withdrawal (2017); NES, surface nanotopography and functional coatings (2020s); and EMNV-coated stents, currently in preclinical development (2021–present). EMNV-coated stents remain experimental and have not yet entered clinical practice. Modified from ref. [10, 11] using Adobe Creative Suite Package [(Illustrator, version 28.7.1 and Photoshop, version 25.12) (Adobe Systems Incorporated, San Jose, CA, USA)]. POBA, plain old balloon angioplasty; BMS, bare-metal stents; DES, drug-eluting stents; NES, nanoengineered stent; EMNV, exosome-mimicking nanovesicle.

The primary goal of technological innovation in coronary stents is to improve mechanical performance, endothelialization, and drug delivery, ultimately enhancing clinical outcomes. Bioinspired platforms integrate nanotechnology and regenerative biology to address unmet clinical challenges such as late ST and delayed vascular healing. The diagram in Fig. 1 (Ref. [10, 11]) outlines the major milestones in coronary stent development, from balloon angioplasty (BA) and BMS to DES, BVS, and the new class of nanoengineered and exosome-mimetic stents.

Plain old balloon angioplasty (POBA), introduced in the late 1970s, offered a nonsurgical method of revascularization, but was unable to overcome the problems of elastic recoil and restenosis. This led to the emergence of BMS in the 1990s, which provided structural support but failed to prevent neointimal hyperplasia (NIH). In the early 2000s, DESs with antiproliferative drug coatings were introduced. However, these also raised concerns about delayed healing and thrombosis. Second-generation DESs, introduced in the early 2010s, improved upon these with biocompatible polymers and more effective drugs, significantly reducing adverse events. The third generation DESs, developed just a few years later from the second-generation ones and characterized by biodegradable polymers and ultra-thin supports, further improved long-term safety and performance. In the years concurrent with third generation DESs, BVS emerged with the promise of temporary support and complete absorption, while in the following years they faced mechanical support and clinical limitations [10, 11].

In recent years, the coronary stent field has begun to shift toward bioinspired stents integrating nanostructured surfaces and exosome-mimetic coatings. The main goal of this innovation is to revitalize natural vascular healing mechanisms through targeted, smart therapeutic application [8, 9].

The issues addressed in stent technologies highlight the importance of striking a balance between mechanical scaffolding and biocompatibility. Elastic recoil has been a significant problem in POBA interventions, and restenosis in BMS interventions. The first generation of DESs reduced restenosis but led to delayed healing and late ST. More advanced platforms (second and third generation DESs) using thinner supports and biodegradable polymers have not fully eliminated this challenge. BVS are designed to restore vascular physiology after resorption but encountered limited radial strength and scaffold thrombosis [12, 13]. These ongoing challenges create a rationale for bioinspired nanoengineered stents and exosome-mimicking platforms.

Below, the important features of the major stent classes that shape interventional cardiology are briefly reviewed [1, 8, 9, 12, 13, 14, 15, 16, 17, 18, 19].

### 2.1 BMS

The platforms of BMSs, such as stainless steel and cobalt-chromium (Co-Cr), provide essential mechanical scaffolding to prevent acute vascular recoil and restenosis in BA. However, they have led to a high rate of ISR, particularly within the first year after implantation, due to vascular injury and the induction of neointimal hyperplasia.

### 2.2 Drug Eluting Stents (DESs)

DESs are stents coated with polymers that release antiproliferative agents, such as sirolimus or paclitaxel, to inhibit smooth muscle cell (SMC) proliferation and reduce neointimal hyperplasia. First-generation DESs significantly decreased rates of neointimal proliferation and restenosis. Subsequent generations of DESs have evolved to have thinner struts, more biocompatible or biodegradable polymers, and improved drug kinetics, resulting in enhanced endothelial healing and a lower risk of late ST. However, even with these advancements, target lesion revascularization (TLR) is still needed at a rate of 1%–2% per year. The main drawbacks of biodegradable stents are the complexity of delivery to the intended site and the increased risk of acute strut fracture due to low mechanical strength, requiring continuous dual antiplatelet therapy (DAPT) for up to two years.

### 2.3 Bioresorbable Vascular Scaffolds (BVSs)

BVS designed to eliminate the long-term presence of permanent metallic implants, reducing the risk of late thrombosis. In theory, BVSs gradually dissolve over time, reducing long-term polymer exposure and restoring native vessel physiology. However, concerns have been raised about their mechanical integrity, inadequate implantation, and higher rates of scaffold thrombosis.

### 2.4 Nanoengineering Stents (NESs)

NESs aim to improve endothelialization and reduce inflammatory responses through nanometer-scale surface-modified coating designs. Unlike traditional DESs, NESs interact with biological systems through structured surfaces and bio-functional coatings. For example, nano-topographies (e.g., nanopillars or grooves) mimic the extracellular matrix, promoting endothelial cell adhesion while inhibiting platelet activation and smooth muscle proliferation. Surfaces coated with agents such as peptides or heparin-mimetics further enhance anti-inflammatory and anti-restenotic effects. Additionally, some NESs have coatings that respond to local cues such as pH or enzymatic activity, leading to a more controlled release of therapeutics.

Given these properties, NESs represent a materials science-driven approach focused on scaffold-tissue interactions. In contrast, EMNV-coated stents, discussed in the following sections, implement a biological coating strategy using vesicle-mimicking nanocarriers for therapeutic delivery. Both are in preclinical stages and represent distinct yet complementary innovation paths.

### 2.5 EMNV-Coated Stents

EMNV-coated stents represent a biologically inspired approach distinct from materials-focused nanoengineering stents. Instead of modifying the scaffold topography, EMNV coatings integrate synthetic vesicles that replicate the lipid bilayer and surface ligands of natural exosomes. This enables site-specific delivery of microRNAs, proteins, and anti-inflammatory mediators directly to the injured vessel wall. Preclinical studies have demonstrated selective release under enzymatic or pH triggers, reduced neointimal growth, and improved endothelial coverage in animal models. Translational development is still in its early stages, and significant challenges remain in large-scale Good Manufacturing Practices (GMP) production, reproducibility of vesicle loading, and long-term safety validation.

Table 1 (Ref. [4, 5, 10, 11, 13, 15, 16, 17, 20, 21, 22]) present a comparative overview of current and emerging stent platforms. The table summarizes their core materials, advantages, and limitations. It also includes ISR, TLR, ST and strut thickness.

**Table 1.** **Comparative features of coronary stent technologies**.

Stent type	Key materials	Key advantages	Key limitations	ISR/TLR rate*	Strut thickness (µm)	Long-term outcomes	References
BMS	Stainless steel, Co-Cr, Pt-Cr alloys	Simple, robust scaffold	High NIH → ISR	~20–30% at 6–12 months	~100–140	Late restenosis common	[10, 11, 16]
1st generation DES	Metal platform (stainless steel or Co-Cr) + durable polymer coatings, with sirolimus/paclitaxel	Potent antiproliferative drugs	Delayed endothelialization; ↑ late ST	~8–12% at 12 months	~120–150	Lower ISR vs BMS; concerns about late ST	[10, 11, 15, 16, 17]
2nd/3rd generation DES	Co-Cr or Pt–Cr alloy + biocompatible/biodegradable polymers, with limus drugs	Thinner supports; better polymers	Remaining delayed healing in high-risk lesions	~5–8% at 12 months	~60–90	Durable efficacy; lower late ST	[4, 10, 16]
BVS	PLLA; magnesium or zinc alloys	Temporary scaffold; vascular restoration	Risk of thrombosis; thicker supports; placement precision	Higher TLR/thrombosis compared to contemporary DES; ISR variable	~150–180	Mixed/less favorable long-term outcomes	[5, 10, 13]
NES	Conventional metal backbone with surface nano-topographies (e.g., titanium nanotubes, nanopillars)	Enhanced endothelialization; reduced platelet adhesion (preclinical)	Experimental; durability and scalability not proven	Preclinical only (qualitative ISR reduction)	Similar to DES	No clinical data	[20, 21]
EMNV covered stents (preclinical)	Conventional metal scaffold (lipid bilayer-based) with surface coating of EMNV	Targeted, pro-healing/anti-inflammatory application	Scalability; short-term data only	Neointimal thickness at 30 days 42%↓ compared to BMS (rat model)	None	No human data; long-term unknown	[22]

*ISR/TLR values ​​represent typical ranges reported in large reviews and meta-analyses rather than device-specific trial results. Results vary depending on lesion complexity, patient population, and procedural technique. Strut thickness values ​​are representative ranges compiled from device reviews; their exact dimensions vary for each stent model. BMS, bare-metal stent; DES, drug-eluting stent; BVS, bioabsorbable scaffold; NES, nanoengineered stent; EMNV, exosome-mimicking nanovesicle; ISR, in-stent restenosis; TLR, target lesion revascularization; ST, stent thrombosis; NIH, neointimal hyperplasia; Co-Cr, cobalt-chromium; Pt-Cr, platinum-chromium; PLLA, poly-L-lactic acid. ↑, indicates increase; ↓, indicates decrease.

As shown in Table 1, successive generations of stents have progressively reduced restenosis rates thanks to thinner struts and improved polymers. However, despite these advances, challenges such as delayed healing, thrombosis, and lack of bioactive integration remain unresolved, providing the rationale for investigating EMNV-coated platforms.

## 3. Biological Challenges in Stent Healing

Understanding the underlying etiology and pathophysiology of ISR is crucial to guide and optimize preventing ISR. Major ongoing challenges include delayed endothelialization, chronic inflammation, neointimal hyperplasia, and late ST.

### 3.1 Delayed Endothelialization

Endothelial healing is critical for thrombosis resistance and vascular healing. Antiproliferative drugs in DESs, especially in the early generations, lead to NIH and increased thrombotic risk. Newer devices have been developed to overcome these limitations, but the issues are still under investigation [14, 15].

### 3.2 Chronic Inflammation

Stent implantation causes endothelial denudation and subsequent activation of the inflammatory foreign body reaction. Additionally, polymers or metal components may trigger hypersensitivity, contributing to chronic inflammation and poor healing [11]. Antiproliferative drugs in DESs, especially in the early generations, lead to NIH and increased thrombotic risk. Long-term DAPT is often required after DES intervention to reduce this risk [14, 15].

### 3.3 Neointimal Hyperplasia

Excessive proliferation of vascular smooth muscle cells may lead to neointimal hyperplasia, the primary cause of ISR. Atherosclerotic changes may occur within the neointima over time, which may contribute to restenosis or thrombosis. Although DES inhibits this process, it may also impair endothelial repair. NIH after stent implantation limits long-term favorable outcomes [16].

### 3.4 Late and Very Late Stent Thrombosis

ST is one of the most feared complications of PCI with high mortality rates. The majority of ST occurs in the acute (first 24 hours) and subacute (>24 hours–30 days) periods (10% to 25%). Persistence of local inflammation after 90 days is associated with a higher risk of delayed endothelialization, ISR, and late or very late ST [11].

Fortunately, recent advances in devices, techniques, and antiplatelet therapies have helped minimize its occurrence (~0.5% to 1%). However, late (>30 days) and very late (>1 year) ST remain rare (0.2%–0.6% per year), but serious events [14, 17].

Understanding and addressing the biological barriers underlying all of these challenges is critical to improving long-term stent performance and patient outcomes. Innovative strategies such as bioactive and biomimetic coatings, including exosome-mimetic platforms, have emerged as approaches to support healing processes aimed at resolving these issues.

## 4. Technological Perspective on Exosome-Mimetic Nanoengineering

Exosomes are nanometer-scale extracellular vesicles (30–150 nm) secreted by various cell types that play an important role in cell-to-cell communication. They contain bioactive molecules such as proteins, lipids, and microRNAs (miRNAs). These molecules allow them to influence the biological function of recipient cells in a highly specific manner. Due to their natural origin and biocompatibility, exosomes have become promising tools for targeted delivery in regenerative medicine and cardiovascular therapy [18, 19]. Exosomes often reflect the properties of the cells from which they originated. For example, platelet-derived exosomes carry growth factors such as vascular endothelial growth factor (VEGF), platelet-derived growth factor (PDGF) and Transforming growth factor β (TGF-β) [6]. Mesenchymal stem cell-derived exosomes are rich in bioactive molecules such as lipids, mRNA, and miRNAs that regulate immune responses, promote angiogenesis, and reduce inflammation [9]. Cardiomyocyte-derived exosomes include heat shock protein 20 (Hsp20), Hsp60, and tumor necrosis factor-α (TNF-α). These molecules specifically promote angiogenesis, improve immune response, and eventually contribute to cardiac remodeling [23].

### 4.1 Exosome-Mimetic Strategies

Exosomes, as natural nanocarriers, hold promise for preventiving ISR by leveraging their biocompatibility, targeted delivery, cell-to-cell communication abilities [24]. However, natural exosomes face limitations such as low yield, heterogeneity, and challenging isolation processes. Engineered EMNVs that mimic the structural and functional properties of natural exosomes provide a scalable and customizable manufacturing approach to overcome these challenges [25]. These mimicry systems can be functionalized to deliver therapeutic miRNAs, anti-inflammatory peptides, and other bioactive agents that regulate vascular healing responses after stent deployment [23].

EMNV can be easily produced with yields 100-fold higher than natural exosomes, making them advantageous for clinical-scale production. They exhibit similar stability, distribution, and immune compatibility to natural exosomes, with are less complexity. Furthermore, they can be modified to improve cellular uptake and targeting properties [26].

### 4.2 Controlled-Release Platforms

To maximize therapeutic efficacy, systems that can provide precise temporal release of bioactive payloads at the site of vascular injury using stimuli-sensitive mechanisms such as pH or enzyme sensitivity can be developed and utilized. To release the payloads (such as drugs or bioactive molecules) to the target site, EMNVs can be placed on biomaterial-based controlled release platforms [26, 27]. This strategy minimizes systemic exposure while enhancing local healing, a critical requirement for new-generation coronary stents. The key benefits of this strategy include increased reendothelialization and decreased NIH [28].

Recently, several preclinical studies have aimed to quantify the performance of exosome coatings sensitive to environmental conditions. Zou *et al*. [22] developed a lipoprotein-associated phospholipase A_2_ (Lp-PLA_2_)-sensitive multivesicle vesicle-coated pro-efferocytic stent that selectively releases exosomes in inflamed vascular environments. *In vitro* tests showed an initial 12% immediate release on day 1 and a cumulative release of approximately 21% on day 14 under PLA_2_ stimulation, whereas baseline conditions resulted in a significantly lower release. Real-time quartz crystal microbalance analyses confirmed that more than 50% of the vesicle content could be released within the first hour of enzyme exposure, confirming the sensitivity of the system [22]. In a complementary *in vivo* experiment using a rat model of atherosclerosis, Zou *et al*. [22] demonstrated both structural and biochemical benefits, with exosome-eluting stent implantation leading to an approximately 42% reduction in neointimal thickness and an approximately 40% reduction in lesional Lp-PLA_2_ concentrations compared to bare-metal stents after 30 days. Enzyme-activated platforms also offer sustained therapeutic release capabilities. For example, hydrogel matrices have provided therapeutic payload delivery lasting 2–4 weeks when activated by disease-associated enzymatic processes, as documented in studies examining laboratory-based release protocols [25]. Meanwhile, the complexities of drug release assessment (including media types, flow dynamics, and device design) have been extensively addressed in the methodological literature on drug-eluting stents [29].

These data demonstrate that stimuli-responsive coatings, including EMNV layers, can be engineered for controlled temporal and spatial release tailored to vascular pathology. Such quantitative and methodological characterization is vital for the translation of these platforms into clinically testable stent technologies.

Appropriately designed nanoscale surface features on stents can enhance the effects of EMNVs by harmonizing them. For example, modifications such as nanopillars or grooves can promote protein adsorption and cellular behavior, reducing thrombosis and inflammation while promoting endothelialization. Such EMNV-coating integration supports selective cellular healing [23, 26].

## 5. Engineering Designs for Bioinspired Stents

**Fig. 2.**
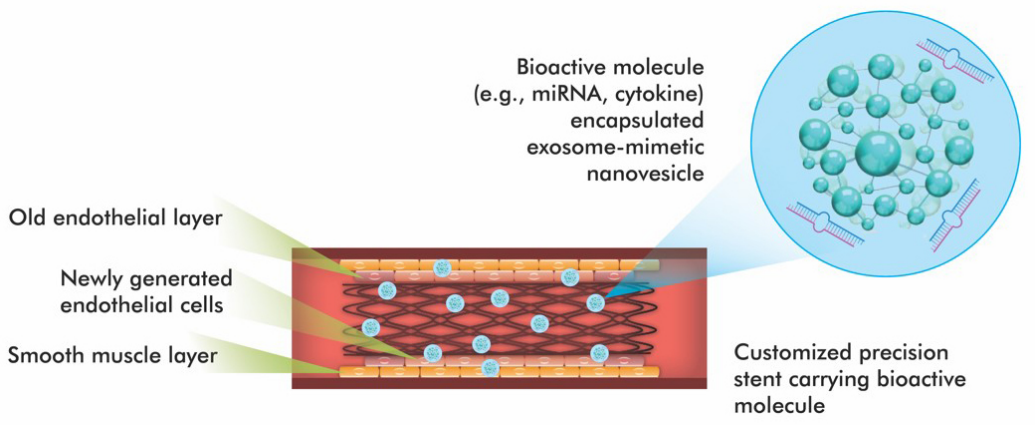
**Schematic design of a new generation coronary stent containing exosome-mimicking nanovesicles (EMNVs)**. Modified from ref. [28] using Adobe Creative Suite Package [(Illustrator, version 28.7.1 and Photoshop, version 25.12) (Adobe Systems Incorporated, San Jose, CA, USA)].

The field of coronary stenting is experiencing unprecedented developments with a significant emphasis on developing new stent surfaces. The primary aim of these innovations is to improve the effectiveness of vascular implants with minimal ISR. For these reasons, they focus on increasing endothelialization and reducing inflammatory responses through surface modifications and novel coatings [20, 30]. Fig. 2, Ref. [28]) highlights the conceptual design of an advanced stent coated with bioactive EMNVs. These nanovesicles are loaded with bioactive agents such as angiogenic miRNAs and anti-inflammatory cytokines. The structure of stent is engineered for site-specific targeting and controlled release in response to environmental signals such as pH, enzyme activity, or oxidative stress. These platforms are primarily designed to enhance endothelial regeneration, reduce NIH and prevent restenosis.

Among the numerous strategies being investigated, stent surface engineering techniques that modify the surface topography, chemistry, and roughness, as well as those that pattern the surface with biologics or drugs, have gained traction. However, the results of these observations have not yet been translated into clinical coronary stent applications [21].

A substantial innovation for the proposed stent design is the integration of EMNV coatings, which are biomimetic structures designed to mimic the structure and function of native exosomes [31]. These synthetic vesicles offer a sophisticated therapeutic payload delivery method that can respond to the local microenvironment of vascular injury. EMNVs have been structurally engineered to mimic critical features of native exosomes that govern cellular recognition and targeted delivery. Their lipid bilayer membranes play a protective role for encapsulated cargoes such as microRNAs and cytokines. These membranes also exhibit a biomimetic interface that efficiently fuses with endothelial cell membranes. Additionally, EMNVs can be functionalized with surface proteins or synthetic ligands that mimic adhesion molecules found in native exosomes, such as integrins or tetraspanins, to enhance selective uptake by endothelial cells at the site of vascular injury [24, 26]. This ligand-mediated adhesion promotes rapid re-endothelialization while reducing nonspecific distribution. When functionalized with platelet-mimicking proteins or peptide sequences, EMNVs can enable platelets to naturally hover on the damaged endothelium, further enhancing local retention and therapeutic efficacy [25]. Together, these structural adaptations enable EMNVs to achieve cell-specific targeting and efficient cargo delivery while minimizing off-target effects. This gives EMNV-coated stents an advantage, which is not available with traditional polymer-based stent coatings.

These EMNVs can be constructed from lipid bilayers, polymeric shells, or nanoparticle-lipid hybrids. They mimic native exosomes in terms of size, surface proteins, and cargo encapsulation [4, 9, 23]. The coatings can be designed to fuse with platelet membranes or incorporate targeting ligands, thereby increasing site-specific adhesion and minimizing systemic distribution [25]. Regulatory miRNAs such as miR-126 (angiogenic and anti-inflammatory) and miR-145 (smooth muscle cell differentiation) can be loaded into vesicles to reprogram vascular healing. Pro-endothelial factors such as VEGF mimics and anti-inflammatory cytokines can further promote rapid endothelialization and immune modulation [24, 26].

Engineered EMNVs can be affected by environmental changes. They can release their contents in response to local pH changes (e.g., acidosis at injury sites), enzymatic activity (e.g., release of matrix metalloproteinases), temperature fluctuations or oxidative stress, which are common signs of vascular injury, thus offering precise spatiotemporal control [9, 27, 28].

These smart, environmentally responsive features enable the targeted release of anti-proliferative, pro-healing or antithrombotic substances only when pathological cues are present. They also minimize off-target effects [27].

The potential clinical significance in overcoming the challenges of traditional drug-eluting stents in preventing late ST and polymer hypersensitivity, accelerating endothelialization, and reducing inflammation is undeniable. They also have the potential to contribute to personalized and regenerative cardiovascular therapies. However, prior to clinical application, proposed stents must undergo rigorous *in vitro* and *in vivo* evaluations to assess mechanical performance, degradation kinetics, hemocompatibility, and bioactivity. Animal models that simulate human vascular pathology are vital to both validate therapeutic potential and inform regulatory strategies [29].

## 6. Clinical Translation and Regulatory Issues

Natural exosomes demonstrate excellent therapeutic effects by encapsulating various regulatory proteins, microRNAs (miRNAs), messenger RNAs (mRNAs), and other naturally active substances. However, the effects of exosomes carrying various functional biomolecules on hosts have not been fully elucidated. Due to these fundamental reasons, injectable exosome therapies have not been approved by regulatory authorities such as the US FDA in the USA and the European Medicines Agency (EMA) in the European Union countries [32].

Similar to native exosomes, the transition of EMNV-coated stents from the lab to clinical use also faces several translational and regulatory challenges. These platforms function as combination products that integrate biologics (miRNAs, cytokines), devices (scaffolds), and drugs (therapeutic payloads), which add complexity to regulatory assessment. These hybrid platforms will likely be classified as drug-device-biologic combination products governed by multi-tiered approval authorities. This necessitates a evidence-based demonstration of biological safety, sterility, reproducibility, and performance in both *in vitro* and *in vivo* environments. For example, EMNV surface proteins and encapsulated bioactive molecules must meet biomarker standards, while the scaffold must pass the International Organization for Standardization (ISO) standards for implantable devices [26, 31, 32].

EMNVs also require reproducible, GMP-compliant production. While EMNVs have the advantage of being produced at up to 100-fold higher yields than native exosomes, batch consistency in cargo loading, EMNV integrity, and targeting performance remains a significant technical hurdle [26, 31]. Ultimately, while these improvements in production efficiency are encouraging, the transition to industrial-scale production remains a critical obstacle. As highlighted by Wang *et al*. [32], regulatory pathways for exosome-derived or exosome-mimicking products face ongoing challenges. Product heterogeneity, difficulties in developing potency assays, and a lack of harmonized international standards for manufacturing and quality control are among these challenges [32]. These concerns directly apply to EMNV-coated stents, where reproducibility and consistency in vesicle production remain key determinants of translational applicability.

The major bottleneck in the translation of EMNV-coated stents lies in the scalability of nanovesicle production. Current production methods, such as extrusion, sonication, or microfluidic synthesis, typically produce heterogeneous vesicle populations with diameters ranging from 50–200 nm. Furthermore, significant batch-to-batch variability in drug loading and membrane protein incorporation occurs [24, 25]. Such heterogeneity can compromise the reproducibility of therapeutic effects and pose challenges for standardization under GMP conditions. Moreover, consistent adhesion and stability of biological layers during large-scale vesicle coating, sterilization, and application of metallic scaffolds are significant challenges. This poses a significant barrier to production, particularly in industrial settings. Addressing these bottlenecks is critical to ensuring EMNV-covered stents move beyond proof-of-concept. While microfluidic and bioreactor-based methods offer promising results, robust process controls, validated efficacy testing, and demonstration of long-term storage stability are still required for regulatory acceptance [33, 34].

Hu *et al*. [8] showed that exosome-coated stents accelerated re-endothelialization and reduced ISR 28 days after implantation compared with DESs and BMSs. However, preclinical validation using advanced animal models simulating human-like vascular pathology is of substantial importance. Studies by Gallet *et al*. [35] have shown that cardiosphere-derived exosomes improve cardiac function and reduce infarct size in porcine models of myocardial infarction.

Preclinical studies have demonstrated promising efficacy with EMNV-coated stents. However, most reports are limited to observation periods of 28–30 days [8, 22]. While these assessments are sufficient to capture early neointimal changes and inflammatory responses, they do not provide information on chronic healing, late thrombosis, or long-term durability of the coating. Traditional DES and BVS platforms have generally been evaluated in preclinical models for ≥90 days before translation. Follow-up in clinical trials has been extended to 12 months or longer [36]. To our knowledge, there are no comprehensive studies addressing outcomes beyond one month with EMNV-covered stents. Before regulatory approval or clinical testing, systematic long-term studies are critical to confirm sustained neointimal control, endothelial healing, and the absence of late adverse events. This period should ideally extend to 3–6 months in large animal models [37].

In the clinical phase, clinical trial design for exosome-mimetic stents also requires careful endpoint selection and patient stratification. Primary endpoints should focus on traditional measures such as target lesion revascularization and major adverse cardiac events, while secondary endpoints may include novel biomarkers of endothelialization and inflammatory response [38].

For regulatory approval, long-term studies evaluating the potential immunogenicity, biodistribution, and clearance pathways of EMNV components are critical. Well-designed studies are required to evaluate clinical efficacy, considering the heterogeneity of patient populations and lesion characteristics. Regulatory evaluation of EMNV-coated stents is complicated by their classification as combination products that integrate a device scaffold with biologically active nanovesicles. In the United States, such products are subject to the FDA’s Office of Combination Products under Title 21 of Code of Federal Regulations (CFR) Part 3, which outlines procedures for assigning primary regulatory responsibility among centers (typically CDRH and CBER) [39]. This requires demonstration of both device safety (e.g., mechanical integrity, delivery reliability) and biological safety (e.g., vesicle potency, immunogenicity, manufacturing consistency). In Europe, the Medical Device Regulation (MDR 2017/745) and the advanced therapy medicinal product (ATMP) guideline mandate a risk-based assessment of new coatings and biodelivery systems [40, 41]. These frameworks emphasize that translation of EMNV-coated stents will require not only clinical evidence but also standardized potency assays, GMP-compliant vesicle manufacturing, and robust long-term safety monitoring.

## 7. Limitations of the Current Landscape and Future Directions

Although exosome-mimetic stent coatings represent a state-of-the-art strategy, their current development is still predominantly in the preclinical stage and faces several limitations.

First and foremost, most studies on stent covered with EMNV are limited to short-term endpoints with long-term biocompatibility, safety, and pharmacokinetics uncertain [23, 28, 29]. Additionally, while synthetic EMNVs have scalable potential, the reproducibility of functional properties such as EMNV size, miRNA loading, fusion efficiency and optimal EMNV dosage has not been sufficiently standardized [31].

The third concern is the mechanical and biological trade-offs. Integrating bioactive EMNV coatings into structurally robust scaffolds affects radial strength, flexibility, and deliverability, especially in bioresorbable platforms such as poly-L-lactic acid or magnesium alloys. The simultaneous optimization of mechanical support and biological deliverability is still under-researched [26, 35]. Another important concern is targeting specificity and off-target effects. Although EMNVs can be designed to respond to environmental stimuli such as pH and temperature changes, there is a risk of nonspecific uptake or uncontrolled release. Fine-tuning is still needed [27].

The final consideration centers on clinical correlation. No EMNV-coated stent has yet entered human clinical trials. The lack of human safety or efficacy data remains a barrier to its clinical application. Despite these challenges, the convergence of bioinspired materials science, nanotechnology, and cardiovascular biology continues to rapidly advance the field. Addressing these limitations and conducting more rigorous and carefully designed studies help establish clinically applicable platforms.

Jiang *et al*. [25] reported that platelet membrane-coated EMNVs significantly reduced plaque size and inflammation in an atherosclerotic mouse model, supporting translational potential. Such observations provide promise for personalized EMNV platform options for cardiovascular stent applications. For example, patient-derived stem cells or platelets can be used to generate autologous exosome mimics that potentially reduce immunogenicity and improve targeting [24].

Artificial intelligence (AI) can be a powerful enabler in the clinical deployment of next-generation coronary stents. AI and machine learning can be applied to optimize EMNVs-cargo combinations, scaffold geometry, and release kinetics based on patient-specific data. AI systems can support various stages of stent development, including biomaterial selection, surface engineering, patient-specific therapeutic strategies, and post-procedural follow-up and clinical decision making [36, 42]. Recent studies offer concrete applications in this regard. Min *et al*. [43] trained a deep learning model on pre-procedural intravascular ultrasound (IVUS) data and achieved a high correlation (r ≈ 0.80) in predicting post-stent lumen area, with under-expansion accuracy reaching 94% (area under curve (AUC) = 0.94). In a complementary approach, Gharaibeh *et al*. [44] used machine learning on optical coherence tomography (OCT) images to predict stent under-expansion in calcified lesions, achieving frame-level accuracy of AUC ≈ 0.85 and root-mean-square-error ≈ 0.04 mm^2^ (r ≈ 0.94). Beyond imaging, recent reviews highlight how computational optimization strategies, such as topology optimization and surrogate modeling, can inform stent scaffold geometry and *in silico* material design [45]. These observations may provide a data-driven basis for EMNV-coated platforms.

As in many other fields, personalized medicine can be expected to shape the future of EMNV-coated stents. Genetic predisposition to CAD and restenosis, combined with AI-assisted pre-procedural planning and stent optimization, may allow for tailoring of vesicle cargo composition, release kinetics, and scaffold geometry to individual patients [6, 36]. 3D bioprinting could enable the fabrication of vascular scaffolds with customized geometries and microarchitectures that can be functionalized with EMNV layers. This could potentially improve both mechanical performance and biological integration [46]. Bioabsorbable alloys such as magnesium and zinc are currently being investigated in next-generation scaffolds [13]. Similarly, these alloys could provide temporary mechanical support while enabling localized, biologically active healing by enabling EMNV release. Such hybrid strategies could accelerate the path to first human trials.

## 8. Conclusion

EMNV-coated stents represent a promising avenue in coronary intervention. These platforms have the potential to address persistent issues of traditional stent platforms, such as restenosis, late thrombosis, and delayed vascular healing. By mimicking the structural and functional properties of native exosomes, EMNVs provide a rational vehicle for targeted drug delivery and localized immunomodulation. Thus, they offer the potential to expand the therapeutic possibilities of stent-based therapy. Preclinical studies demonstrate reduced neointimal growth and favorable effects on inflammation, though evidence remain short-term. However, the transition to clinical application is still in its early stages.

Major hurdles include the scalability and reproducibility of EMNV production, the stability of biocoatings during storage and distribution, and the classification of these platforms by regulatory agencies such as the FDA and EMA. Overcoming these challenges requires harmonized manufacturing standards, validated efficacy testing, and robust long-term safety testing. However, it’s important to note that, unlike traditional drug-eluting stents, EMNV-coated devices combine a mechanical scaffold with a biologically active component. This requires regulatory placement under combination product frameworks that require evidence of both mechanical safety and biological stability.

When evaluating existing data, one of the concrete priorities that emerges is the need for rigorously designed large animal studies and early-phase human trials to confirm safety and efficacy in heterogeneous patient populations. Second, advances in microfluidic and bioreactor technologies can be used to improve large-scale EMNV production under GMP conditions. Third, the synergy between 3D bioprinting of vascular scaffolds and bioresorbable alloy platforms could help accelerate translation. Finally, the integration of AI-assisted design and imaging analytics could enable personalized stent selection and more objective assessment of device performance.

Overall, EMNV-covered stents, while still in the preclinical phase, have the potential to become transformative platforms in interventional cardiology if the scientific, manufacturing, and regulatory hurdles are systematically addressed.

## Abbreviations

AI, Artificial intelligence; BMS, Bare-metal stent; BVS, Bioabsorbable vascular structure; CAD, Coronary artery disease; DES(S), Drug-eluting stent(s); EMA, European Medicines Agency; EMNV, Exosome-mimicking nanovesicle; FDA, US Food and Drug Administration; GMP, Good Manufacturing Practices; ISR, In-stent restenosis; NES, Nanoengineered stent; NIH, Neointimal hyperplasia; PCI, Percutaneous coronary intervention; ST, Stent thrombosis; TLR, Target lesion revascularization.
